# A network analysis of peritraumatic dissociation and subsequent intrusive memories

**DOI:** 10.1080/20008066.2025.2555793

**Published:** 2025-12-02

**Authors:** Fiona Maccallum, Hannah Gutmann, Mark Creamer, Meaghan O'Donnell, David Forbes, Alexander C. McFarlane, Derrick Silove, Richard A. Bryant

**Affiliations:** a School of Psychology, University of Queensland, Brisbane, Australia; b School of Psychology, University of New South Wales, Sydney, NSW, Australia; c Department of Psychiatry, Phoenix Australia, University of Melbourne, Melbourne, Australia; d Discipline of Psychiatry, Adelaide Medical School, Adelaide, Australia; e School of Psychiatry, University of New South Wales, Sydney, NSW, Australia

**Keywords:** Peritraumatic dissociation, PTSD, re-experiencing symptoms, network analysis, traumatic injury, Trastorno de estrés postraumático, disociación, análisis en red, longitudinal

## Abstract

**Background:** Cognitive models of posttraumatic stress disorder (PTSD) propose that disturbed processing during a traumatic event results in a distinctive encoding style, which contributes to the subsequent occurrence of re-experiencing symptoms.

**Objective:** Despite this claim, there is a lack of evidence regarding the distinctive relationship between peritraumatic dissociation and subsequent re-experiencing symptoms.

**Method:** This study used a longitudinal sample of traumatic injury survivors (*n* = 443) who were assessed during hospital admission and within one month of trauma exposure for peritraumatic dissociation (using the Peritraumatic Dissociative Experiences Questionnaire), and subsequently 382 (86.2%) participants were re-assessed three months later for PTSD (using the Clinician-Administered PTSD Scale). Network analyses were applied to determine the relationships between peritraumatic dissociation and each of the DSM-IV PTSD symptoms three months later.

**Results:** Eighty-two (9.5%) patients met criteria for PTSD at three-months post injury. There were strong positive edges between peritraumatic dissociation and subsequent amnesia, as well as the re-experiencing symptoms of physical reactivity to reminders, flashbacks, intrusions and dreams, and to a lesser extent emotional numbness and hypervigilance.

**Conclusions:** The finding that peritraumatic dissociation is related to subsequent re-experiencing symptoms more strongly than other PTSD symptoms is consistent with cognitive models that emphasize the role of aberrant encoding of a traumatic event in the etiology of PTSD re-experiencing symptoms.

## HIGHLIGHTS

- Theory predicts peritraumatic dissociation impacts encoding of trauma memories.
- Network analysis showed that dissociation after trauma is specifically associated with subsequent reexperiencing symptoms.
- Findings suggest that peritraumatic dissociation may disturb encoding of trauma memories.

## Abstract (es)

**Antecedentes:** Los modelos cognitivos del trastorno de estrés postraumático (TEPT) proponen que el procesamiento alterado durante un evento traumático resulta en un estilo de codificación distintivo, lo cual contribuye a la aparición posterior de síntomas de reexperimentación.

**Objetivo:** A pesar de esta afirmación, no existe evidencia alguna acerca de la relación distintiva entre disociación peritraumática y síntomas de reexperimentación posteriores.

**Método:** Este estudio utilizó una muestra longitudinal de sobrevivientes de lesiones traumáticas (*n* = 443) que fueron evaluados durante su hospitalización y dentro del mes de la exposición al trauma para detectar disociación peritraumática (utilizando el Cuestionario de Experiencias Disociativas Peritraumáticas), y posteriormente 382 participantes (86,2%) fueron reevaluados 3 meses después para TEPT (utilizando la Escala de TEPT Administrada por el Clínico). Se aplicaron análisis de redes para determinar la relación entre disociación peritraumática y cada uno de los síntomas de TEPT del DSM-IV tres meses después.

**Resultados:** Ochenta y dos (9.5%) pacientes reunía criterios para TEPT a los tres meses después de la lesión. Se observaron fuertes diferencias positivas entre disociación peritraumática y amnesia posterior, así como entre los síntomas de reexperimentación de reactividad física a los recordadores, flashbacks, intrusiones y sueños, y en menor medida, embotamiento emocional e hipervigilancia.

**Conclusiones:** El hallazgo que la disociación peritraumática se relacionan con síntomas de reexperimentación posterior mas fuertemente que otros síntomas de TEPT es consistente con los modelos cognitivos que enfatizan el papel de la codificación aberrante de un evento traumático en la etiología de los síntomas de reexperimentación del TEPT.

Intrusive memories are central to posttraumatic stress disorder (PTSD) in that most people with the disorder report these symptoms in the forms of unwanted memories, nightmares, or flashbacks (Bryant, O'Donnell, et al., 2011). Cognitive models of PTSD posit that intrusive memories are predominantly a function of distinct encoding patterns during trauma, in which excessive arousal results in fragmented encoding (often referred to as data-driven processing) that limits the extent to which mental representations of the trauma are embedded in autobiographical memory (Ehlers & Clark, 2000). It is proposed that trauma memories intrude spontaneously because they are readily activated by related stimuli within the memory network (Foa et al., 1989). This perspective has conceptual similarities with developments in cognitive neuroscience that attempt to explain the capacity to discriminate between newly encoded memories from previously stored memories, referred to as mnemonic discrimination. Specifically, when memories are encoded in a way that they are not clearly differentiated from other memories in terms of their detail or context, they are more likely to overlap with each other, which may lead to ready activation of irrelevant memories because of connections with many other representations (Kheirbek & Hen, 2014; Kheirbek et al., 2012).

There is much evidence that data-driven processing of trauma information is associated with subsequent PTSD (Ehlers et al., 1998; Ehring et al., 2008). There is also evidence that this form of encoding predicts subsequent intrusive memories (Halligan et al., 2002). Fragmented encoding of traumatic events can also be conceptualized as a form of peritraumatic dissociation, which is also predictive of subsequent PTSD symptoms (Murray et al., 2002; Shalev, Freedman, Peri, Brandes, Sahara, et al., 1998). People with PTSD also have poorer mnemonic discrimination than those without PTSD (Bernstein et al., 2020) and people with more severe anxiety have poorer discrimination during stress (Bernstein, 2019). This convergent evidence suggests that fragmented encoding associated with peritraumatic dissociation may contribute to subsequent intrusive symptoms, and in this sense may represent a potential mechanism to explain the relationship between peritraumatic dissociation and later PTSD.

One means to test the relationship between peritraumatic dissociation and subsequent intrusive memories is through network analyses. This statistical approach has attracted much attention in recent years because it presumes that each symptom (or node) is causally linked to other symptoms in the network via relationships that can impact each other (via edges) (Borsboom, 2013; McNally, 2021). That is, rather than assuming that symptoms emerge from a latent disease, the constituent symptoms of a disorder are causally related. For example, in the case of PTSD, nightmares may contribute to sleep problems, which may in turn lead to anger and concentration deficits (McNally, 2015). Network analyses of longitudinal data allows delineation of the links between symptom networks in the peritraumatic phase and their relationship with subsequent symptoms (Bryant et al., 2016). In the wake of the attention to network analyses, many studies have been published regarding the interrelationships of PTSD symptoms (for a review, see Birkeland et al., 2020).

In this study we examined the relationship between peritraumatic dissociation and subsequent intrusive symptoms by applying a network analytic approach to determine the extent to which peritraumatic dissociation is differentially related to subsequent intrusive symptoms relative to other PTSD symptoms. We hypothesized that peritraumatic dissociative reactions would be most strongly associated with re-experiencing symptoms including intrusive memories, as well as psychological and physiological reactivity to reminders, flashbacks, and nightmares.

## 1. Method

### 1.1. Participants

This was conducted as secondary analyses of existing data, and so study design, hypotheses, and analysis plan were not preregistered. The current study drew participants from the Australian Injury Vulnerability Study (IVS), which recruited consecutive injury survivors admitted to four level one trauma centres across Australia between April 2004 and April 2006. The inclusion criteria were people aged 18–70 years, proficient in English, and were admitted to hospital for at least 24 h following traumatic injury. The exclusion criteria included moderate or severe head injury, current psychosis or active suicidality, temporary visitors to Australia, cognitive impairment, or under police guard.

There were 443 trauma patients (336 males; 75.8%) of mean age 38.0 ± 13.5 years who met inclusion criteria and completed the baseline assessment. Participants had survived transport accidents (298, 67.3%), assaults (28, 6.3%%), traumatic falls (60, 13.5%), work injuries (28, 6.3%), or other traumatic injuries (29, 6.6%). The mean time between traumatic injury and assessment was 6.2 ± 7.3 days. There were 180 (40.6%) patients who had sustained a mild traumatic brain injury. There were 382 patients (86.2%) who completed the subsequent assessment three-months after the initial baseline measures.

### 1.2. Procedure

The study was approved by the human research ethics committees at the participant hospitals at the participating hospital, and all participants provided written informed consent. Research assistants conducted interviews at least 24 h after the traumatic injury. The mean duration between injury and initial assessment was 6.17 days (SD = 7.32). Peritraumatic dissociation was assessed using the Peritraumatic Dissociative Experiences Questionnaire (PDEQ; Marmar et al., 1997) The PDEQ is a 10-item self-report scale that indexes a range of perceptual and cognitive dissociative experiences in the context of a traumatic event; in this study the PDEQ had strong internal consistency (α = .86). Three months after the traumatic injury, participants were followed up by research psychologists who conducted an assessment of PTSD via telephone interviews using the Clinician Administered PTSD Scale based on DSM-IV criteria (CAPS; Blake et al., 1995). All assessments were audio-recorded to ensure ongoing adherence to the protocol. For each symptom a severity score was calculated as the sum of the CAPS frequency and intensity scores, resulting in items with values ranging from 0-8; the internal consistency of the CAPS was strong (.88).

#### 1.2.1. Data analysis

Network estimation: A network of regularized partial correlation coefficients was computed in R version 4.0.1 (R Core Team, 2018) using the GLASSO procedure from the R package *qgraph* (v 1.9.1 Epskamp et al., 2012). In a regularized partial correlation network, each edge represents the partial correlation between two nodes after controlling for all other variables in the dataset. The weight of the edge represents the strength of the partial correlation. Thicker edges represent larger partial correlations. When the partial correlation is zero, no edge is drawn between the nodes. The GLASSO procedure employs a ‘least absolute shrinkage and selection operator’ (LASSO) correction to shrink very small connections to zero. The degree of correction is determined by minimizing the Extended Bayesian Information Criteria. The result is a more parsimonious network in which fewer edges are used to explain variation in the data (Epskamp, Borsboom, & Fried 2018; Epskamp & Fried, 2018; Friedman, Hastie, & Tibshirani, 2008). Two participants had missing responses on the PDEQ. Pairwise deletion of missing data was employed. The accuracy and stability of the network were assessed using the R package *bootnet* (Epskamp et al., 2018). *Bootnet* computes 95% confidence intervals around each edge and tests for significant differences in edge weights and node strength (Epskamp et al., 2018). Node strength represents the absolute sum of the edge weights leading into a single node. It provides an indication of the potential influence or importance of that node within the network. The package also produces an estimate, known as the *CS-coefficient,* which indicates the degree to which observed differences in node strengths are stable and thus interpretable. A value greater than .50 is considered interpretable (Epskamp et al., 2018). The CS-coefficient in the current analysis was .516.

## 2. Results

### 2.1. Posttraumatic stress levels

At 3-month follow-up, 39 (10.2%) participants met criteria for PTSD. Table 1 presents the mean severity for each symptom at follow-up.

**Table 1.** Mean posttraumatic stress symptom severity scores for peritraumatic and 3-month assessments.

Symptom	Peritraumatic	3-Month
Intrusions	1.28±2.28	1.13±2.01
Nightmares	.77±1.89	.63±1.67
Flashbacks	.49±1.46	.48±1.28
Distress to reminders	1.12±2.10	1.06±1.88
Physiological reactivity	.87±1.94	.92±1.83
Avoidance of thoughts	1.23±2.25	1.11±2.08
Avoidance of reminders	.22±1.02	.88±2.03
Amnesia	2.55±3.31	2.40±3.37
Disinterest in activities	.52±1.40	.84±1.88
Detachment	.47±1.42	.91±2.05
Numbing	.36±1.24	.74±1.83
Foreshortened future	.21±.95	.78±1.55
Sleep disturbance	4.09±3.06	2.54±2.99
Anger	1.43±2.13	1.47±2.27
Concentration deficits	1.41±2.12	1.30±2.23
Hypervigilance	.57±1.47	1.49±2.22
Startle response	.63±1.59	.80±1.76
Total CAPS	18.21±16.67	19.29±21.90

### 2.2. Network analyses of peritraumatic dissociation and subsequent PTSD symptoms

**Figure 1.**
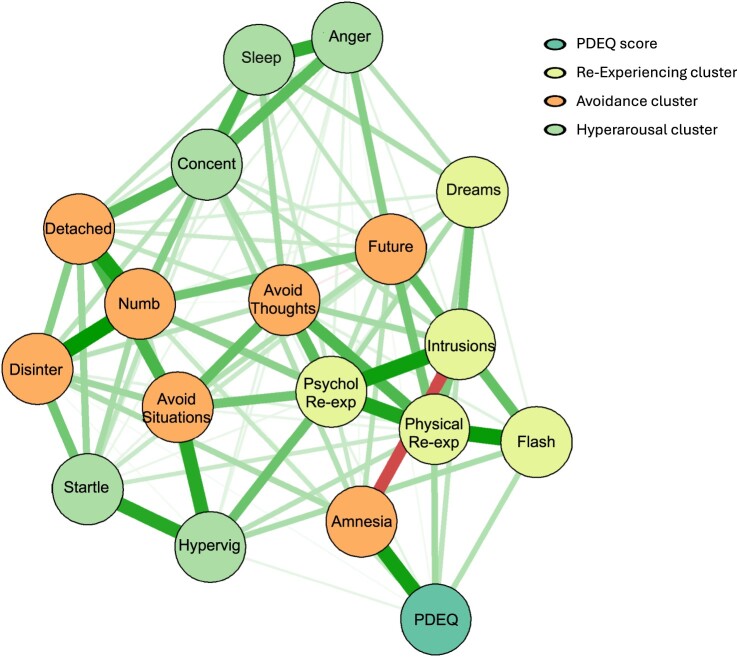
Regularized partial correlation network of peritraumatic dissociation and PTSD symptoms assessed 3-month later. Nodes represent symptom and edges represent partial correlation between the symptoms, after controlling for all other correlations of a given node. Edge thickness scaled against maximum absolute weight across two networks. Intrus: intrusions; Dream: nightmares; Flash: flashbacks; Psyc_R: psychological reactivity; Phys_R: physiological reactivity; Avoid_T: avoidance of thoughts; Avoid_S: avoidance of situations; Amnesia: amnesia; Detach: detachment from others; Disint: disinterest in activities; Numb: emotional numbing; Future: foreshortened future; Sleep: sleep disturbance. Anger:  irritability; Vigil: hypervigilance; Concent: concentration deficits; Startle: startle response; PDEQ:  Peritraumatic Dissociative Experiences Questionnaire.

Figure 1 presents the estimated network comprising PTSD items and the total scale score on the PEDQ. To aid visual interpretation, PTSD symptom clusters are shaded differently. As can be seen, the strongest edges were between symptoms within each PTSD cluster. All edges in the network were positive, with the exception of the edge between Amnesia and Intrusions (numeric weights are presented in Supplementary Table 1). Results from the *Bootnet* analysis indicated that only the thickest and thinnest edges were reliably different from each other (see supplementary Figures S1 for 95% confidence intervals), indicating that caution should be taken when interpreting visual differences in edge strengths. The PDEQ was positively connected with the PTSD symptom of amnesia. This edge was one of the strongest in the network and indicated that higher levels of dissociation at baseline were linked with endorsement of greater amnesia at follow-up (see supplementary Figure S2 for test of edge weight differences). There were also positive edges between PDEQ and the re-experiencing symptoms of physical reactivity to reminders, flashbacks. *Bootnet* results indicated that psychological reactivity to reminders and physical reactivity were the two strongest PTSD nodes in the network, followed by emotional numbness and concentration difficulties. This indicated that these nodes had the largest absolute sum of edge weights and therefore were potentially the most influential in the network.

## 3. Discussion

As hypothesized, peritraumatic dissociation was positively associated with the re-experiencing symptoms of intrusive memories, and physiological reactivity to reminders, flashbacks, and nightmares. This pattern of findings can be considered in the context of previous findings that peritraumatic dissociation predicts subsequent PTSD (Murray et al., 2002; Shalev, Freedman, Peri, Brandes, Sahar, et al., 1998). The observation that peritraumatic dissociation was particularly related to re-experiencing symptoms rather than other PTSD symptoms (except for amnesia) extends current knowledge because it highlights a distinct association between peritraumatic dissociation and later re-experiencing. The specific mechanisms underpinning the predictive role of peritraumatic dissociation on subsequent PTSD may involve memory processes. Specifically, the disruptive influence of peritraumatic dissociation on encoding during the traumatic event.

This pattern of findings can be interpreted in the context of cognitive models that propose that data-driven processing, which often involves fragmented and disorganized encoding, can lead to re-experiencing symptoms of PTSD (Ehlers & Clark, 2000). Previous studies have shown that data-driven processing, as measured by scales that index perceptually-based encoding, are predictive of later intrusive memories (Ehlers et al., 1998; Ehring et al., 2008; Halligan et al., 2002). The current finding also accords with evidence that PTSD is characterized by diminished mnemonic discrimination (Bernstein et al., 2020), although we note that the evidence for this proposal is very mixed (Brewin & Fields, 2024; Taylor et al., 2022). Although the current design not permit us to make causal inferences regarding the proposal that fragmented encoding can interfere with the consolidation of trauma memories into a coherent autobiographical narrative of the traumatic experience and contribute to re-experiencing symptoms, these findings are consistent with this proposal. Cognitive models that emphasize the role of perceptual processing in the etiology of re-experiencing symptoms note that elevated arousal during encoding interferes with normal episodic processing of the event (Brewin et al., 1996; Holmes, 2010). The current study did not index arousal during encoding, and so we are not able to clarify the potential role of arousal contributing to the alterations in processing that were associated with later re-experiencing. In the context of evidence that peritraumatic derealization mediates the relationship between peritraumatic arousal and later PTSD symptoms (Bryant, Brooks, et al., 2011) and that arousal can elicit dissociation in the post-trauma period (Nixon & Bryant, 2005a; Nixon et al., 2005b), it is important for future studies to understand how arousal in the peritraumatic phase impacts intrusive symptoms specifically.

The finding that peritraumatic dissociation was associated with subsequent amnesia, which was one of the strongest edges, is not surprising. This association can be explained by several factors. First, dissociative amnesia is one phenomenon measured on the PDEQ, and so it may not be surprising that these two variables were associated. Second, dissociative responses are often associated with avoidance, which can be one factor leading to a person to inhibit retrieval of trauma memories (Cavicchioli et al., 2021). Third, if a person has disturbed encoding at the time of trauma, then the events surrounding the traumatic event are less likely to be subsequently recalled because they have not been adequately consolidated into memory. Strictly speaking, if an event is not encoded then failure to retrieve it does not qualify as dissociative amnesia because the latter involves an inability to access memories that were encoded (Bryant, 2007). Fourth, poor memory of a trauma can occur because of mild traumatic brain injury (which is common following traumatic injuries; Bryant et al., 2010), alcohol intoxication, prescribed medication whilst in hospital; each of these factors can also impede awareness and may be reflected in higher scores on the PDEQ even though they do not necessarily involve dissociative processes. Although DSM-5 explicitly excludes dissociative amnesia when the lack of recall can be attributed to brain injury, medication, or intoxication, measures such as the PDEQ or the CAPS can inadvertently capture these responses as dissociative, and therefore inflate the observed association between amnesia and dissociation.

We note that peritraumatic dissociation was measured approximately one week after the trauma, and although the questions attempted to index reactions that occurred at the time of the trauma, it is not possible to isolate the exact timing of the altered processing to the encoding phase versus the subsequent memory consolidation period. Dissociation that persists over time tends to have a stronger association with PTSD symptoms than transient dissociative reactions (Briere et al., 2005; Panasetis & Bryant, 2003). In DSM-IV peritraumatic dissociation was defined in the context of acute stress disorder as occurring either at the time of the trauma or in the subsequent acute period, and in DSM-5 it is defined without reference to the specific timeframe. This is potentially problematic because it confounds the encoding, consolidation, and retrieval phases of trauma memories (Bryant & Harvey, 1997). There is a need for future work to identify the extent to which altered processing during the traumatic event, as distinct from the subsequent memory consolidation and reconsolidation periods (Monfils & Holmes, 2018), impacts subsequent re-experiencing symptoms. This issue is underscored by the finding that interfering with perceptual processing during the period after encoding, can reduce subsequent intrusive memories (Iyadurai et al., 2018).

The association between peritraumatic dissociation and subsequent emotional numbing accords with conceptualizations of numbing as serving a function of inhibiting emotional states (Frewen & Lanius, 2006). It is feasible that cognitive and emotional processes in the peritraumatic phase that involve impaired processing of emotions promotes a process that becomes chronic emotional numbing. The relationship between peritraumatic dissociation and later hypervigilance may be attributed to dissociation occurring in more severe stress responses or to predisposing characteristics that increase risk for later PTSD. This finding is consistent with cognitive models which explain that PTSD is maintained when individuals experience an ongoing sense of serious, current threat. This occurs when the trauma memory is processed in a data driven manner, without important contextual and temporal information, and when individuals interpret cues or reminders of the trauma in an overly negative way (Ehlers and Clark, 2000). These mechanisms may result in greater hypervigilance at three-months follow-up.

It is interesting that a positive edge exists between peritraumatic dissociation and physiological reactivity. This pattern appears inconsistent with the proposal that dissociative responses are characterized by inhibition of psychophysiological arousal (Lanius et al., 2018). Evidence from studies of healthy individuals support this proposal with low dissociative individuals (Danbock et al., 2021), as well as evidence that dissociative PTSD individuals display inhibited arousal at psychophysiological (Bryant et al., 2000; Griffin et al., 1997) and neural (Lanius et al., 2010) levels. Specifically, dissociative PTSD is associated with diminished physiological reactivity when people are presented with trauma reminders (Griffin et al., 1997). However, there are conflicting findings that dissociation is not associated with reduced physiological arousal (Danbock et al., 2024; Griffin et al., 2002; Kaufmann et al., 2002; Nixon et al., 2005b). Recent reviews have indicated that there is not a robust relationship between dissociation and suppressed arousal or reactivity (Beutler et al., 2022; Roydeva & Reinders, 2021). Instead, the association between dissociation and arousal is complex and underpinned by multiple factors (Bryant, 2007). In this context the current finding of a moderate relationship between peritraumatic dissociation and physiological reactivity is not surprising.

We note several methodological limitations of the study. First, the sample comprised traumatic injury survivors, the majority of which were caused by motor vehicle accidents. There is greater prevalence of dissociative responses following severe and interpersonal trauma (Zatzick et al., 1994), thus the current finding needs to be replicated with other trauma groups. Relatedly, most of the population was resilient after their traumatic injury, and future studies could usefully address this issue in populations with higher rates of PTSD. Second, we did not index other peritraumatic factors which may contribute to dissociation, including arousal and distress (Fikretoglu et al., 2007), and which may subsequently influence data-driven processing and re-experiencing symptoms. We also note that we assessed dissociation via self-report rather than structured interview, which may not provide an optimal measure of dissociative responses. Further, follow-up assessments were administered by telephone, raising potential doubts as to the validity of the outcomes. However, this format has been shown to yield comparable responses to in-person interviews (Aziz & Kenford, 2004). Despite these limitations, this study advances our current knowledge of the development of intrusive PTSD symptoms by pointing to the specific relationship between peritraumatic dissociative responses and subsequent re-experiencing phenomena. Network analyses should be further utilized to refine how other peritraumatic trauma reactions, including arousal and data-driven processing, can interact in the emergence of persistent PTSD symptoms.

## Supplementary Material

Dissociation supplementary_3.docx

## Data Availability

Access to the de-identified data is available at https://www.10.6084/m9.figshare.23650608.
