# Supplementary material for: A network analysis of peritraumatic dissociation and subsequent intrusive memories
Source: Eur J Psychotraumatol. 2025 Dec 2;16(1):2555793. doi: 10.1080/20008066.2025.2555793 (PMC12673980; doi:10.1080/20008066.2025.2555793)
Supplement: Dissociation supplementary_3.docx [file ZEPT_A_2555793_SM4218.docx]

-Table 1: Network Edge weights

pdeq_sum BIntrude BDreams BFlash BPsyReact BPhysReact

pdeq_sum 0.000000000 0.06373520 0.03708997 0.073249291 0.00000000 0.08685615

BIntrude 0.063735203 0.00000000 0.13373228 0.101520927 0.22487161 0.12403973

BDreams 0.037089972 0.13373228 0.00000000 0.024618601 0.07852538 0.08071372

BFlash 0.073249291 0.10152093 0.02461860 0.000000000 0.02155181 0.23108794

BPsyReact 0.000000000 0.22487161 0.07852538 0.021551811 0.00000000 0.21213241

BPhysReact 0.086856151 0.12403973 0.08071372 0.231087935 0.21213241 0.00000000

BAvThought 0.000000000 0.08498157 0.00000000 0.030589040 0.15493082 0.16610269

BAvSit 0.000000000 0.00000000 0.07274082 0.022102514 0.13212520 0.00000000

BAmnesia 0.221681844 -0.16931241 0.00000000 0.000000000 0.00000000 0.00000000

BDisint 0.010720191 0.00000000 0.00000000 0.029509297 0.00000000 0.00000000

BDetach 0.009555873 0.00000000 0.03562276 -0.002955811 0.00000000 0.00000000

BNumb 0.053717017 0.00000000 0.01965390 0.000000000 0.10867092 0.00000000

BFuture 0.000000000 -0.02357665 0.00000000 0.145047692 0.06492209 0.13060799

BSleep 0.000000000 0.05883198 0.07725857 0.001478624 0.00000000 0.00000000

BAnger 0.012907330 0.02572486 0.06715423 0.000000000 0.02847108 0.00000000

BConcent 0.026589472 0.06667988 0.00000000 0.000000000 0.08464408 0.00000000

BVigil 0.022359911 0.00000000 0.02512650 0.080163316 0.13781627 0.06681814

BStartle 0.000000000 0.00000000 0.05146499 0.000000000 0.01989973 0.05174146

BAvThought BAvSit BAmnesia BDisint BDetach BNumb

pdeq_sum 0.000000000 0.00000000 0.22168184 0.01072019 0.009555873 0.053717017

BIntrude 0.084981567 0.00000000 -0.16931241 0.00000000 0.000000000 0.000000000

BDreams 0.000000000 0.07274082 0.00000000 0.00000000 0.035622761 0.019653904

BFlash 0.030589040 0.02210251 0.00000000 0.02950930 -0.002955811 0.000000000

BPsyReact 0.154930824 0.13212520 0.00000000 0.00000000 0.000000000 0.108670923

BPhysReact 0.166102691 0.00000000 0.00000000 0.00000000 0.000000000 0.000000000

BAvThought 0.000000000 0.14553931 0.00000000 0.06589822 0.058639080 0.008529685

BAvSit 0.145539311 0.00000000 0.00000000 0.09020602 0.170193643 0.000000000

BAmnesia 0.000000000 0.00000000 0.00000000 0.07856737 0.000000000 0.000000000

BDisint 0.065898224 0.09020602 0.07856737 0.00000000 0.111456680 0.238007491

BDetach 0.058639080 0.17019364 0.00000000 0.11145668 0.000000000 0.219403833

BNumb 0.008529685 0.00000000 0.00000000 0.23800749 0.219403833 0.000000000

BFuture -0.019348557 0.02103677 0.06817360 0.00000000 0.049934629 0.133661149

BSleep 0.089328468 0.00000000 0.05295535 0.00000000 0.060817507 0.000000000

BAnger 0.022891564 0.00000000 0.01301304 0.00000000 0.040866847 0.025951722

BConcent 0.047184030 0.00000000 0.07023138 0.06633142 0.157628231 0.114060022

BVigil 0.022726945 0.20154537 0.00000000 0.01191272 0.002419482 0.061587541

BStartle 0.024699565 0.00000000 0.00000000 0.13144292 0.092878104 0.083081782

BFuture BSleep BAnger BConcent BVigil BStartle

pdeq_sum 0.00000000 0.000000000 0.01290733 0.02658947 0.022359911 0.00000000

BIntrude -0.02357665 0.058831983 0.02572486 0.06667988 0.000000000 0.00000000

BDreams 0.00000000 0.077258570 0.06715423 0.00000000 0.025126504 0.05146499

BFlash 0.14504769 0.001478624 0.00000000 0.00000000 0.080163316 0.00000000

BPsyReact 0.06492209 0.000000000 0.02847108 0.08464408 0.137816269 0.01989973

BPhysReact 0.13060799 0.000000000 0.00000000 0.00000000 0.066818140 0.05174146

BAvThought -0.01934856 0.089328468 0.02289156 0.04718403 0.022726945 0.02469957

BAvSit 0.02103677 0.000000000 0.00000000 0.00000000 0.201545366 0.00000000

BAmnesia 0.06817360 0.052955354 0.01301304 0.07023138 0.000000000 0.00000000

BDisint 0.00000000 0.000000000 0.00000000 0.06633142 0.011912721 0.13144292

BDetach 0.04993463 0.060817507 0.04086685 0.15762823 0.002419482 0.09287810

BNumb 0.13366115 0.000000000 0.02595172 0.11406002 0.061587541 0.08308178

BFuture 0.00000000 0.015213735 0.11153899 0.04681304 0.000000000 0.00000000

BSleep 0.01521374 0.000000000 0.19348333 0.16850845 0.000000000 0.03440350

BAnger 0.11153899 0.193483328 0.00000000 0.15466496 0.027046129 0.02157711

BConcent 0.04681304 0.168508450 0.15466496 0.00000000 0.000000000 0.06182104

BVigil 0.00000000 0.000000000 0.02704613 0.00000000 0.000000000 0.20220638

BStartle 0.00000000 0.034403499 0.02157711 0.06182104 0.202206377 0.00000000

Supplementary Figure 1: Graphical representation of network edge Confidence intervals produced by Bootnet.


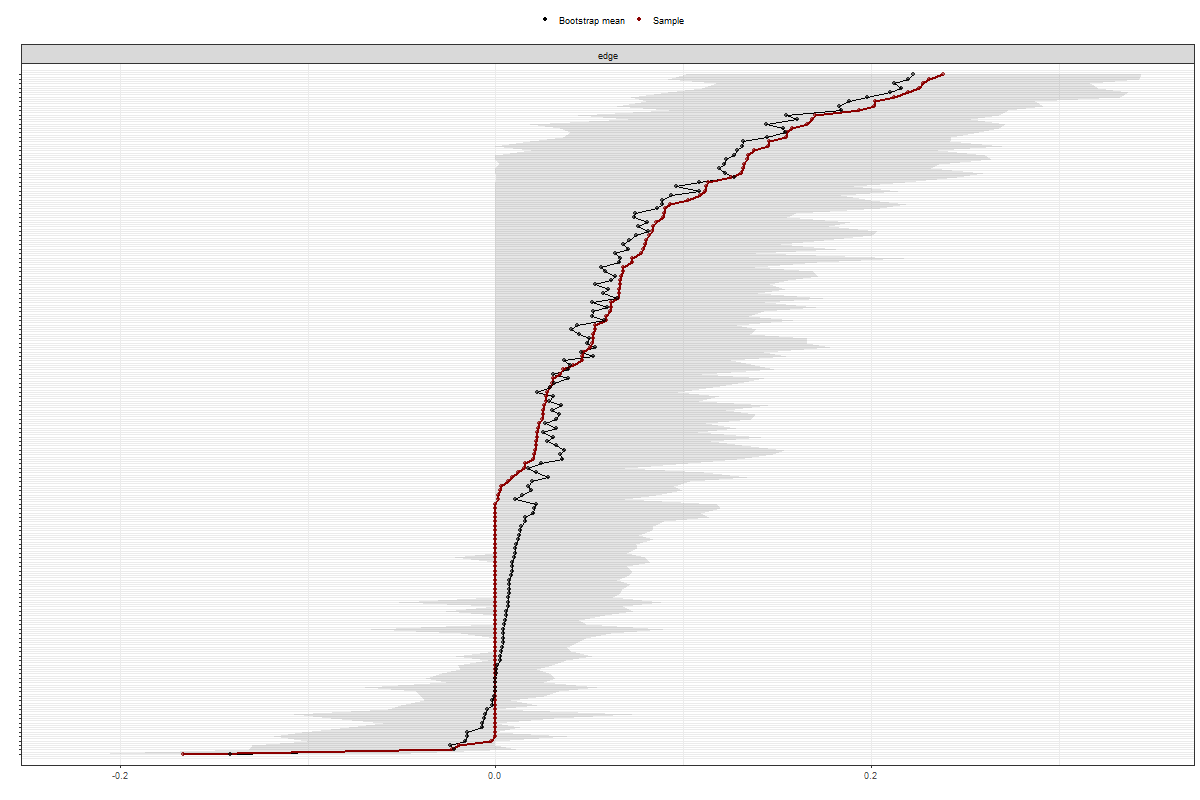


Supplementary Figure 2: Results from Bootnet test of edge differences
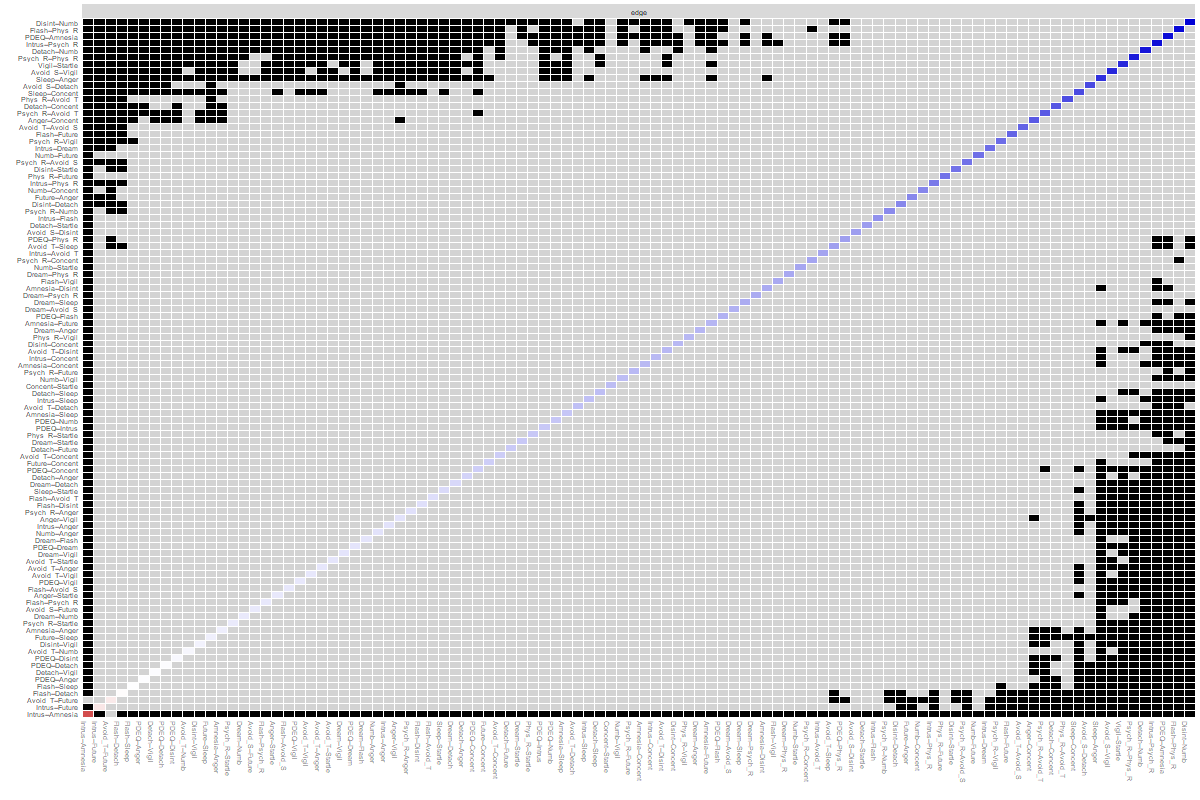


Note: Black boxes indicated significant differences (significant level p<.05)

Supplementary Figure 1: Results from Bootnet test of node strength differences


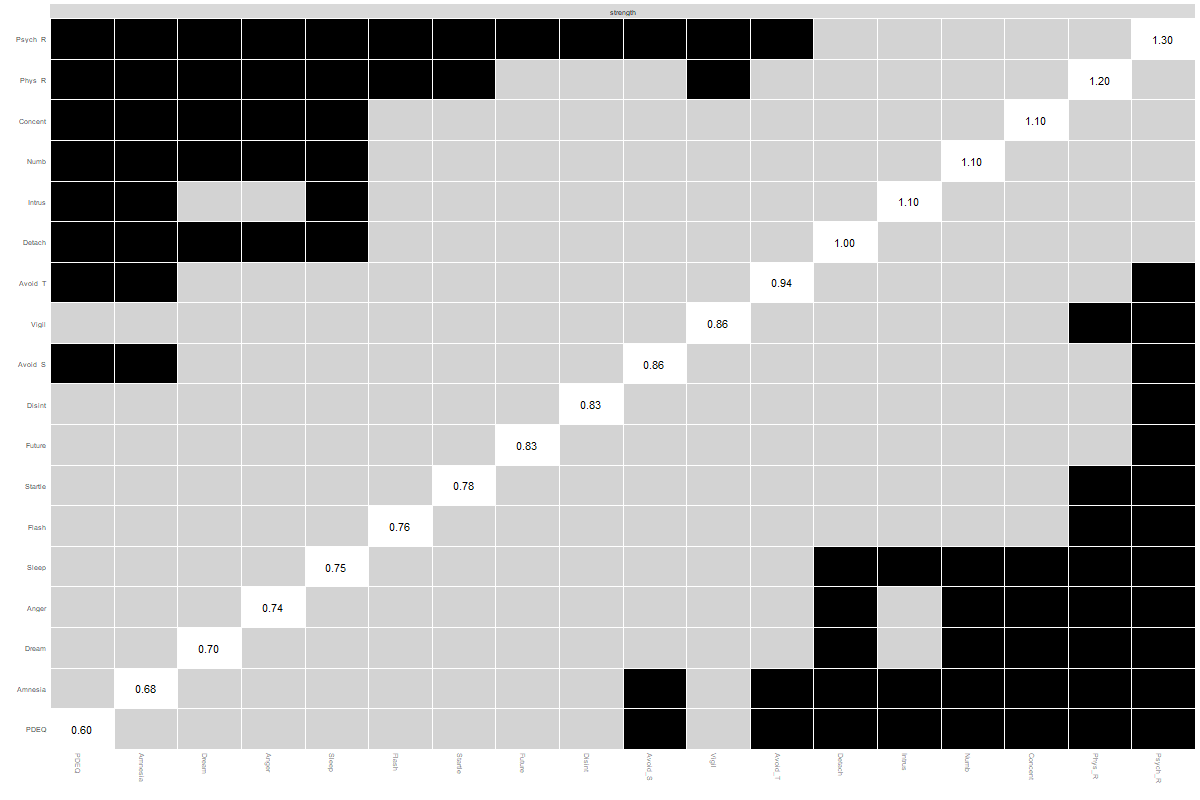


Note: Black boxes indicated significant differences (significant level p<.05)

R syntax

#A Network Analysis of Peritraumatic Dissociation and Subsequent Intrusive Memories

#TO CLEAR ENVIRONMENT

rm(list=ls())

#LOADING PACKAGES

library("qgraph")

library("bootnet")

############# DATA LOADING and PREPARATION ########################

#set working directory

setwd (insert file location)

#dissociation and time 2 PTSD

x1 <- read.csv("DissociationIntrusion_manuscript_final.csv", header=TRUE, na=999)

#change to numeric (asalasso requires numeric)

x1 <- as.data.frame(lapply(x1,as.numeric, na.rm=TRUE))

#Select columns

d2 <-subset(x1, select = 11:28)

###################grouping and labels for GRAPH##########################

group_labels <- list (

"PDEQ score" = c(1),

"Re-experiencing cluster" = c(2, 3, 4, 5, 6),

"Avoidance cluster" = c(7, 8, 9, 10, 11, 12, 13),

"Hyperarousal cluster" = c(14, 15, 16, 17, 18))

group_cols <- c("#66C2A5", "#E6F598", "#FDAE61","#ABDDA4")

node_ls <- c("PDEQ", "Intrusions", "Dreams", "Flashbacks",

"Psychol Reexp", "Physical Reexp", "Avoid Thoughts",

"Avoid Situations", "Amnesia", "Disinterest",

"Detachment", "Numbness", "Future",

"Sleep", "Anger", "Concentration",

"Hypervigilance", "Startle")

wrapped_labels <- sapply(node_ls, function(label) {

paste(strwrap(label, width = 10), collapse = "\n")

})

##########SYNTAX NETWORK####################################

#correlation matrices ASSUMPTION CHECKS

c1p <- cor(d2, method = "pearson", use='pairwise.complete.obs')

c1s <- cor(d2, method = "spearman", use='pairwise.complete.obs')

corc <- cor(c1s[lower.tri(c1s)], c1p[lower.tri(c1p)], method="spearman") # = 0.982168

print(corc)

#OK TO USE PEARSON#

######################PTSD and dissociation network ########################

glasso0 <- EBICglasso(c1p, n=442)

graph_EBIC_L <- qgraph(glasso0,lambda.min.ratio = 0.025,

layout = "spring", groups=group_labels,

color = group_cols,

labels= wrapped_labels, label.cex = 3,

label.scale.equal =TRUE, label.prop = .4, minimum = .049,

legend = TRUE,

legend.cex = 0.4)

#############################save figure

tiff(file="PTSDandDissoc_REV300_L.tiff",width = 2400, height = 2000, res = 300)

plot(graph_EBIC_L)

dev.off()

#############################save edge weights

sink(file="PTSDandDissoc_edgeweights.txt", append = TRUE)

print(glasso0)

sink()

######################### NETWORK RELIABILITY #################

BootNet1db <- estimateNetwork(d2,default = "EBICglasso",

tuning = .25)

graphBoot1db <- plot(BootNet1db,

layout = "spring", groups=groups2,

color = group_cols,

labels= node_ls,label.cex = 3,

label.scale.equal =TRUE, label.prop = .4, minimum = .049,

legend = TRUE)

Boot1db <- bootnet(BootNet1db)

Boot1dCSb <- bootnet(d2, missing = "pairwise",

default = ("EBICglasso"), tuning = .25,

type =("case"),

statistics = c("strength", "closeness","betweenness"))

######save output as files #################

png(file="PTSDandDissoc_bootCI.png",width=1200, height=800, res = 300)

plot(Boot1db, labels = FALSE, order = "sample", title = "CI")

dev.off()

png(file="PTSDandDissoc_bootCS.png",width=1200, height=800, res = 300)

plot(Boot1dCSb, title = "CS")

dev.off()

png(file="PTSDandDissoc_bootdiff.png",width=1200, height=800, res = 300)

plot(Boot1dCSb, "edge", plot = "difference", onlyNonZero = TRUE,

color = "gray", order = "sample", title = "dense")

dev.off()

png(file="PTSDandDissoc_bootstrength.png",width=1200, height=800, res = 300)

plot(Boot1d, "strength", order = "sample", title = "dense")

dev.off()

sink(file="PTSDandDissoc_bootcs.txt", append = TRUE)

corStability(Boot1dCSb)

sink()
